# Death With Function and Graft Failure After Kidney Transplantation: Risk Factors at Baseline Suggest New Approaches to Management

**DOI:** 10.1097/TXD.0000000000001273

**Published:** 2022-01-13

**Authors:** Massini A. Merzkani, Andrew J. Bentall, Byron H. Smith, Xiomara Benavides Lopez, Matthew R. D’Costa, Walter D. Park, Walter K. Kremers, Naim Issa, Andrew D. Rule, Harini Chakkera, Kunam Reddy, Hasan Khamash, Hani M. Wadei, Martin Mai, Mariam P. Alexander, Hatem Amer, Aleksandra Kukla, Mireille El Ters, Carrie A. Schinstock, Manish J. Gandhi, Raymond Heilman, Mark D. Stegall

**Affiliations:** 1 Division of Nephrology and Hypertension, Mayo Clinic, Rochester, MN.; 2 William J. von Liebig Center for Transplantation and Clinical Regeneration, Mayo Clinic, Rochester, MN.; 3 Division of Clinical Trials and Biostatistics, Mayo Clinic, Rochester, MN.; 4 Division of Nephrology and Hypertension, Mayo Clinic, Scottsdale, AZ.; 5 Division of Transplant Surgery, Mayo Clinic, Scottsdale, AZ.; 6 Division of Nephrology and Hypertension, Mayo Clinic, Jacksonville, FL.; 7 Division and Laboratory Medicine and Pathology, Mayo Clinic, Rochester, MN.; 8 Division of Transfusion Medicine, Mayo Clinic, Rochester, MN.; 9 Department of Surgery, Mayo Clinic, Rochester, MN.; 10 Department of Immunology, Mayo Clinic, Rochester, MN.

## Abstract

**Background.:**

Improving both patient and graft survival after kidney transplantation are major unmet needs. The goal of this study was to assess risk factors for specific causes of graft loss to determine to what extent patients who develop either death with a functioning graft (DWFG) or graft failure (GF) have similar baseline risk factors for graft loss.

**Methods.:**

We retrospectively studied all solitary renal transplants performed between January 1, 2006, and December 31, 2018, at 3 centers and determined the specific causes of DWFG and GF. We examined outcomes in different subgroups using competing risk estimates and cause-specific Cox models.

**Results.:**

Of the 5752 kidney transplants, graft loss occurred in 21.6% (1244) patients, including 12.0% (691) DWFG and 9.6% (553) GF. DWFG was most commonly due to malignancy (20.0%), infection (19.7%), cardiac disease (12.6%) with risk factors of older age and pretransplant dialysis, and diabetes as the cause of renal failure. For GF, alloimmunity (38.7%), glomerular diseases (18.6%), and tubular injury (13.9%) were the major causes. Competing risk incidence models identified diabetes and older recipients with higher rates of both DWFG and nonalloimmune GF.

**Conclusions.:**

These data suggest that at baseline, 2 distinct populations can be identified who are at high risk for renal allograft loss: a younger, nondiabetic patient group who develops GF due to alloimmunity and an older, more commonly diabetic population who develops DWFG and GF due to a mixture of causes—many nonalloimmune. Individualized management is needed to improve long-term renal allograft survival in the latter group.

## Abstract 2

Supplemental Digital Content is available in the text.

## INTRODUCTION

Long-term outcomes after kidney transplantation have improved little over the past 2 decades despite improvements in patient management tools. including HLA antibody testing, polyoma virus (BK) testing, and greater consensus regarding Banff histologic classifications.^1^ Many studies of long-term graft loss have focused on the role of alloimmunity in “death-censored graft loss” (ie, graft loss not due to death, termed simply graft failure [GF] here); however, relatively few studies have examined risk factors for nonalloimmune causes of GF in detail, and even fewer have studied GF and death with a functioning graft (DWFG) together. The latter is important because DWFG and GF are competing events. Identifying specific populations of patients with different risk factors for different types of graft loss is crucial to individualized patient management.

The goal of this study was to assess risk factors for specific causes of graft loss to determine to what extent patients who develop either DWFG or GF (ie, graft loss due to causes other than death) have similar baseline risk factors for graft loss.

## MATERIALS AND METHODS

### Study Population

This was a retrospective cohort study of all kidney transplant recipients occurring from January 1, 2006, and December 31, 2018, at the 3 Mayo Clinic sites (Minnesota, Florida, and Arizona). The last follow-up data were May 10, 2020. We excluded recipients who had received a nonrenal organ transplant in addition to the kidney, had a positive crossmatch (B or T lymphocyte), were ABO-incompatible, or did not consent to be included in research. The electronic medical record was used to retrieve data in this Mayo Clinic Institutional Review Board approved study. The posttransplant course was assessed by a combination of clinical, laboratory parameters and histology, including both surveillance and biopsies for cause. Graft and patient statuses were registered by using the electronic medical records and a dedicated transplant database from Mayo Clinic. Up-to-date graft status and patient status were confirmed using site-specific United Network Organ Sharing STAR data. More detailed methods are presented in the Supplemental Methods (SDC, http://links.lww.com/TXD/A398). Diabetes here refers to diabetes as the assigned cause of renal failure at the time of transplantation. Not all patients had native kidney biopsies proving this. Posttransplant diabetes was not a variable in our assessments.

### Endpoint and Definition of the Cause of Graft Loss

The primary endpoints were the incidence and causes of DWFG (defined as the allograft was still functioning when the recipient died) and GF (defined as return to chronic dialysis or retransplantation). We employed an adjudication system for GF causes in which 2 transplant nephrologists independently assigned causes into 6 major categories (surgical complications, alloimmune, glomerular disease, tubular injury, BK, and other). A third transplant nephrologist, blinded to the assigned causes of the first 2 nephrologists, was asked to adjudicate in case of the first 2 disagreed.

### Statistical Analyses

Data are presented as mean (± SD) or median (interquartile range) depending on whether the data were approximately normally distributed. The continuous clinical characteristics were compared by *t* test or Kruskal-Wallis test for variables that were not approximately normally distributed. Categorical variables were compared using the chi-square test. Data were censored at the patients’ last follow-up or May 10, 2020. All statistical tests were 2 sided. *P* values 0.05 or less were considered statistically significant. Univariate and multivariable Cox regression was used to model the incidence of DWFG and GF. Cause-specific Cox regression analysis was performed to model the cause of graft loss for alloimmune and renal tubular injuries and for DWFG due to cardiac, infection, and malignancy. In this case, patients are censored for all causes of death or graft loss except for the specific cause of failure of interest for comparison. This method was chosen to focus on etiology rather than subdistribution hazard estimates.^2-4^ Cumulative incidence curves were generated using the competing risks extension of Kaplan-Meier incidence estimates. Differences in rates between groups were tested using Gray’s extension of the log-rank test for competing risks through the “cmprsk” package in R.^5^ Differences between competing risks were compared based upon the area under the competing risk cumulative incidence curves using a jackknifed estimate of the variance in the difference.

For specific causes of GF, to account for limited power, univariate models were fit first followed by multivariable models with statistically significant variables (*P* < 0.05) at the univariate level. Cross-sectional analysis of the different GF comparing alloimmune as a referent causes using a *t* test or using chi-square for proportion. Note that these proportions and tests are conditioned on an event happening and on evaluating baseline variables prior or during the event. Differences in absolute risk for several predictor variables were estimated using the Aalen-Johansen estimator of incidence, which adjusts predictions from the cause-specific Cox regression for competing risks.

To explore the overlap between patients at greater risk of DWFG and GF, we used the linear predictors from these cause-specific Cox models. Median values for both the DCGF and GF were used to form “quadrants” of low and high predicted risk. JMP PRO version 14 software and R Statistical Program version 3.6.2 were used for statistical analysis.

## RESULTS

### Patients

The study population included 5752 consecutive kidney transplants (Table 1). The mean recipient age was 53.8 ± 13.9 y, 24.9% (1432) were ≥65 y old, 61.1% (3514) were men, 66.2% were White, 13.5% were Black, 9.5% were Hispanic, and 10.8% were of other ethnic groups or unknown. At the time of transplantation, 69.8% were on dialysis, and 10.3% had received a prior kidney transplant; 50.8% received a kidney from a deceased donor, and 98.5% received tacrolimus as part of their maintenance immunosuppression.

**TABLE 1.** Demographics of recipient cohort at the time of transplantation

Baseline characteristics	Total population
	(n = 5752)
Age (y), mean (SD)	53.8 ± 13.9
Male, n (%)	3514 (61.1)
Recipient BMI pretransplant (kg/m^2^), median [IQR]	28.7 [24.7–33.3]
Race, n (%)	
White	3807 (66.2)
Black race	776 (13.5)
Hispanics	546 (9.5)
Others	623 (10.8)
Pretransplant cause of ESRD, n (%)	
Glomerulonephritis	1522 (26.5)
Diabetes mellitus	1376 (23.9)
Hypertensive nephrosclerosis	701 (12.2)
Cystic diseases	688 (12.0)
Retransplant	594 (10.3)
Uropathy	180 (3.1)
Unknown/others	691 (12.0)
Dialysis pretransplant, n (%)	4012 (69.8)
Prior kidney transplant, n (%)	594 (10.3)
cPRA ≥ 80% (n = 4941)^*a*^	528 (10.7%)
Living donor transplant, n (%)	2827 (49.2)
Donor age (y), n (%)	42.2 ± 14.6
Donor male, n (%)	2904 (50.5)
Induction, n (%)	
Alemtuzumab	2449 (42.6)
Thymoglobulin	1932 (33.6)
Anti-CD25	1367 (23.8)
None	4 (0.07)
Maintenance immunosuppression, n (%)^*b*^	
Tacrolimus, MMF, and prednisone	3064 (53.3)
Tacrolimus, MMF	2600 (45.2)
Others	88 (1.5)
Maintenance prednisone^*c*^	3120 (54.2%)
DGF^*d*^	1267 (22.0%)

^*a*^cPRA was calculated using data from UNOS STAR information available in 4941 patients.

^*b*^Maintenance immunosuppressant used in the first 4 mo or last censored day prior the first 4 mo.

^*c*^Chronic maintenance of prednisone as an immunosuppressant after induction.

^*d*^Delayed graft functioning defined as requiring dialysis the first week posttransplant.

BMI, body mass index; cPRA, calculated panel reactive antibody; DGF, delayed graft functioning; ESRD, end-stage renal disease; IQR, interquartile range; MMF, mycophenolate mofetil.

The median follow-up was 3.5 y (2.0–6.4 y). At the time of censoring, 8.0% (462) had >10 y of follow-up, 27.7% (1593) were between 5 and 10 y, and 64.3% (3697) had <5 y of follow-up after transplant. Overall graft loss occurred in 21.6% (1244), including DWFG in 12.0% (691) and GF in 9.6% (553); 78.4% (4508) had a functioning allograft at last follow-up. The most recent follow-up data for patients with a functioning allograft were within 1 y of the censoring date in 62.3% (2808) (Figure S1 and Table S1, SDC, http://links.lww.com/TXD/A398).

### DWFG

The causes of DWFG included malignancy 20.0% (138), infection 19.7% (136), cardiac disease 12.6% (87), and unknown 37.0% (256) (Table 2). Of the 691 cases of DWFG, 12.3% (85) occurred within 1 y of transplantation, 45.4% (314) between 1 and 5 y, and 42.3% (292) >5 y after transplantation.

**TABLE 2.** DWFG after solitary kidney transplantation (2006–2018)

Cause	Time after kidney transplantation			
	Total	<1 y	1–5 y	>5 y
All DWFG	691 (12.0%)	85 (12.3%)	314 (45.4%)	292 (42.3%)
Malignancy	138 (20.0%)	10 (11.8%)	66 (21.0%)	62 (21.2%)
Infection	136 (19.7%)	29 (34.1%)	63 (20.1%)	44 (15.1%)
Cardiac	87 (12.6%)	11 (12.9%)	32 (10.2%)	44 (15.1%)
Other	74 (10.7%)	20 (23.5%)	31 (9.9%)	23 (7.9%)
Unknown	256 (37.0%)	15 (17.6%)	122 (38.9%)	119 (40.8%)

The causes of DWFG are listed by cause and by the time that they occurred with respect to the kidney transplantation.

DWFG, death with a functioning graft.

### Multivariable Analysis of DWFG

**FIGURE 1.**
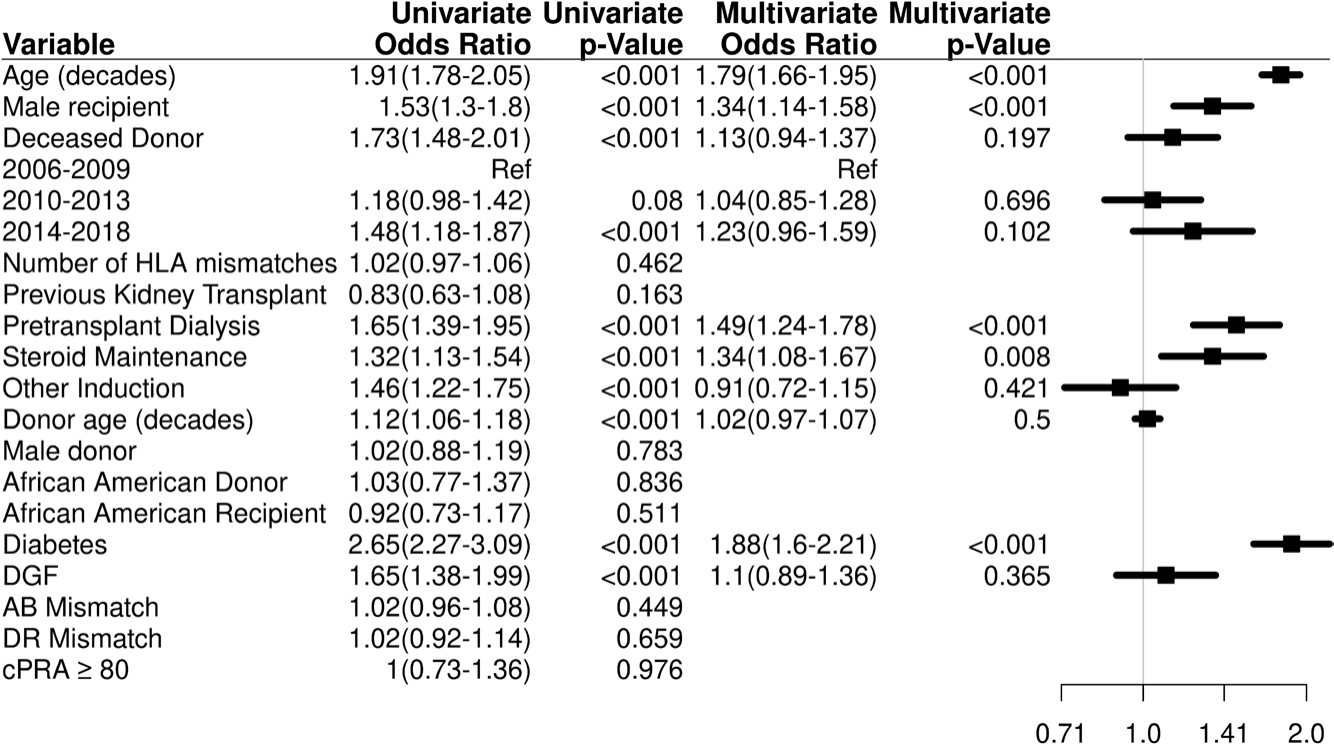
Multivariable analysis for DWFG. Other induction is comparing alemtuzumab vs Thymoglobulin and anti-CD25. Anti-CD25 induction use mostly was basiliximab, and only 2 patients used daclizumab. Diabetes is defined as diabetes mellitus as a cause pretransplant of ESRD. Adjusted for recipient age (10 y), sex, deceased donor, period of transplant, induction, dialysis pretransplant, prednisone therapy, delayed graft functioning, diabetes mellitus as a cause pretransplant of ESRD, and transplant site (Table S10, SDC, http://links.lww.com/TXD/A398). Risk factors for DWFG are increased age, male sex, pretransplant dialysis, and diabetes mellitus as the cause of renal failure and use of prednisone. cPRA, calculated panel reactive antibody; DGF, delayed graft functioning; DWFG, death with a functioning graft; ESRD, end-stage renal disease.

Independent predictors of DWFG are included as follows: older recipient age (hazard ratio [HR] = 1.79; 95% confidence interval [CI], 1.66-1.95; *P* < 0.001), recipient of male sex (HR = 1.34; 95% CI, 1.14-1.58; *P* < 0.001), dialysis pretransplant (HR = 1.49; 95% CI, 1.24-1.78; *P* < 0.001), diabetes mellitus as a cause of end-stage renal disease (ESRD) (HR = 1.88; 95% CI, 1.6-2.21; *P* < 0.001), and prednisone use as maintenance therapy (HR = 1.34; 95% CI, 1.08-1.67; *P* = 0.008) (Figure 1; Table S2, SDC, http://links.lww.com/TXD/A398).

Risk factors for specific causes are included as follows: (1) for malignancy, older age (HR = 1.79; 95% CI, 1.51-2.12; *P* < 0.001), recipient male sex (HR = 1.81; 95% CI, 1.22-2.67; *P* = 0.003), and dialysis at the time of transplant (HR = 1.64; 95% CI, 1.1-2.44; *P* = 0.015; (2) for infection, older age (HR = 1.93; 95% CI, 1.59-2.33; *P* < 0.001), dialysis at the time of transplant (HR = 1.75; 95% CI, 1.14-2.68; *P* = 0.011), diabetes mellitus as a cause of ESRD (HR = 1.55; 95% CI, 1.07-2.23; *P* = 0.020), and prednisone as maintenance therapy (HR = 1.81; 95% CI, 1.13-2.91; *P* = 0.013); and (3) for cardiac, older age (HR = 1.57; 95% CI, 1.28-1.92; *P* < 0.001) and diabetes mellitus as the cause of native renal failure (HR = 2.69; 95% CI, 1.74-4.18; *P* < 0.001) (Tables S3, S4, and S5, SDC, http://links.lww.com/TXD/A398).

The cause of DWFG was unknown in 256 of cases; however baseline characteristics and major risk factors for DWFG (age, diabetes, pretransplant dialysis) were similar in the patients with known and unknown causes of death (Table S6, SDC, http://links.lww.com/TXD/A398).

### GF

Of the 553 GFs, 23.7% (131) occurred in the first year after transplantation, 42.5% (234) between 1 and 5 y, and 33.8% (187) >5 y after transplantation (Table 3). Using our adjudication scheme, the 2 experts agreed in 83.5% (462/553) of cases (Table S7, SDC, http://links.lww.com/TXD/A398). Alloimmunity (214, 38.7%), glomerular diseases (103, 18.6%), and renal tubular injury (77, 13.9%) were the major medical causes of GF (Table 3). In the first year after transplantation, surgical complications and primary nonfunction of the allograft caused 60.3% (79) of graft losses. Alloimmunity was the cause of graft loss in 49.8% (117) of those lost before death in the 1- to 5-y period and 43.3% (81) in the >5-y period (Figure S2, SDC, http://links.lww.com/TXD/A398).

**TABLE 3.** Causes of graft failure by time after kidney transplantation

Cause	Time after kidney transplantation			
	Total	<1 y	1–5 y	>5 y
Total	553 (100%)	131 (23.7%)	235 (42.5%)	188 (33.8%)
Alloimmune	214 (38.7%)	16 (12.2%)	117 (49.8%)	81 (43.3%)
Glomerular diseases	103 (18.6%)	18 (13.7%)	41 (17.4%)	44 (23.5%)
Renal tubular injuries	77 (13.9%)	12 (9.2%)	41 (17.4%)	24 (12.8%)
Primary dysfunction/surgical	79 (14.3%)	79 (60.3%)	0 (0.0%)	0 (0.0%)
BK nephropathy	24 (4.3%)	4 (3.1%)	10 (4.3%)	10 (5.3%)
Unknown/Other	56 (10.1%)	2 (1.5%)	26 (11.1%)	28 (15.0%)
Number at risk at the beginning of the time period	5752	5752	5396	3716

Graft failure (not due to death) by category was determined by an adjudication process in which 2 or more expert nephrologists determined the cause based on chart review. The table also shows the causes of graft loss with respect to time after kidney transplantation and the number of patients followed at the beginning of the time period.

BK, polyoma virus.

Of the 214 cases of alloimmune-mediated graft loss, chronic active antibody-mediated rejection (AMR) (33.2%) was the leading cause of graft loss, followed by mixed acute cellular and active AMR (25.7%), mixed acute cellular and chronic active AMR (19.6%), acute or chronic active cellular rejection alone (20.6%), and active AMR alone (0.9%). Thus, 79.4% of the alloimmune losses were associated with some aspect of antibody-mediated damage (Table S8, SDC, http://links.lww.com/TXD/A398).

The 103 cases of glomerular disease mediating GF included recurrence of primary glomerular disease (59.2%), de novo glomerular disease (9.7%), diabetic nephropathy (14.6%), and thrombotic microangiopathy not related to AMR (16.5%). For the recurrent glomerular disease subgroup (n = 61), the most common disease was primary focal segmental glomerular sclerosis (28, 45.9%), followed by IgA nephropathy (12, 19.7%) and membranoproliferative glomerulonephritis (8, 13.1%).

The renal tubular injuries category has not usually appeared in the literature as a cause of GF. This accounted for 77 (13.9%) GFs including recurrent episodes of acute tubular necrosis due to infection (39.0%), recurrent/chronic hypovolemia (14.3%), a severe episode of acute tubular injury (16.9%), and cardiorenal syndrome (15.6%). GFs due to surgical complications were most commonly due to renal vascular thrombosis (57%) and primary nonfunction (35.4%).

GF due to BK nephropathy (24, 4.3%) was due equally to active BK and inactive BK. Of the 56 GFs due to unknown/other causes, 69.6% (39) had no clinical history or histology necessary to determine the cause of loss, 3 had severe arteriolar hyalinosis, and 2 had malignancy in the renal allograft.

### Multivariable Analysis of GF

**FIGURE 2.**
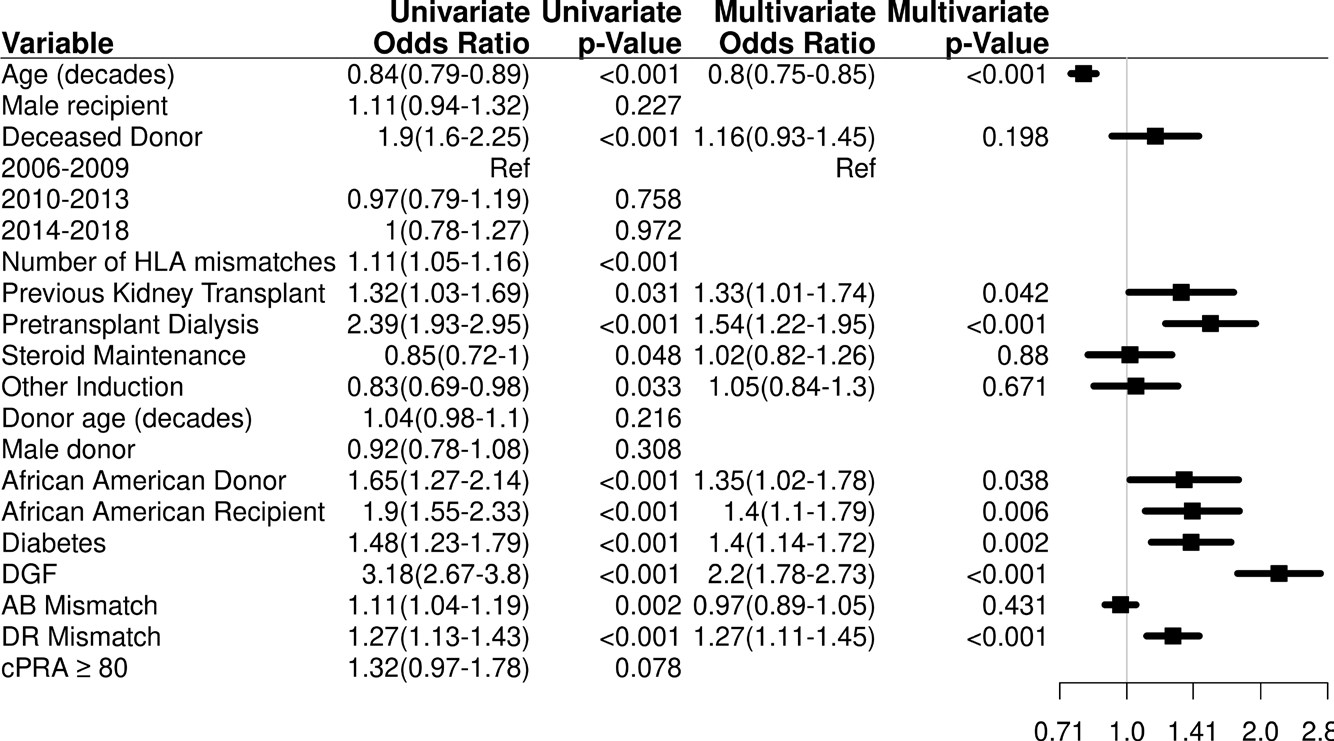
Multivariable analysis for GF (not due to death). Other induction is comparing alemtuzumab vs Thymoglobulin and anti-CD25. Anti-CD25 induction use mostly was basiliximab, and only 2 patients used daclizumab. The use of diabetes is defined as diabetes mellitus as a cause pretransplant of ESRD. Adjusted for age (10 y), deceased donor, HLA A, B mismatch, HLA DR mismatch, induction, dialysis pretransplant, prednisone therapy, prior kidney transplant, donor race, recipient race, delayed graft functioning, diabetes mellitus as a cause pretransplant of ESRD, and transplant site (Table S7, SDC, http://links.lww.com/TXD/A398). Risk factors for GF are increased younger age, prior kidney transplant, pretransplant dialysis, African-American donor and recipient, diabetes mellitus as the cause of renal failure, and HLA DR mismatch. cPRA, calculated panel reactive antibody; DGF, delayed graft functioning; ESRD, end-stage renal disease; GF, graft failure.

In a multivariable model for overall GF (Figure 2; Table S9, SDC, http://links.lww.com/TXD/A398), risk factors were younger recipient age in decades (HR = 0.80; 95% CI, 0.75-0.85; *P* < 0.001), a history of previous kidney transplant (HR = 1.33; 95% CI, 1.01-1.74; *P* = 0.042), dialysis at the time of transplantation (HR = 1.54; 95% CI, 1.22-1.95; *P* < 0.001), Black recipient race (HR = 1.40; 95% CI, 1.1-1.79; *P* = 0.006), diabetes as a cause of ESRD (HR = 1.40; 95% CI, 1.14-1.72; *P* = 0.002), HLA DR mismatch (HR = 1.27; 95% CI, 1.11-1.45; *P* < 0.001), Black donor race (HR = 1.35; 95% CI, 1.02-1.78; *P* = 0.038), and delayed graft function (HR = 2.20; 95% CI, 1.78-2.73; *P* < 0.001).

Analysis of patients who lost their grafts because of different causes showed that, compared with patients with alloimmune causes of GF, these patients with nonalloimmune causes of GF were older, had fewer rejection episodes, and were less likely to be nonadherent (Table 4).

**TABLE 4.** Cross-sectional clinical characteristics of the different cause of GF

Clinical characteristics	Alloimmunen = 214 (Reference group)	Glomerular diseasen = 103		Renal tubular injuries n = 77		BK nephropatHy n = 24		Unknown/Othern = 56	
	n (%), OR mean (SD)	n (%), OR mean (SD)	*P*	n (%), OR mean (SD)	*P*	n (%), OR mean (SD)	*P*	n (%), OR mean (SD)	*P*
Age of the recipient at donation	44.7 ± 14.9	48.9 ± 14.2	0.02	58.1 ± 11.2	<0.001	54.7 ± 14.2	0.002	51.6 ± 14.6	0.002
Sex (male)	121 (56.5)	71 (68.9)	0.03	47 (61.0)	0.46	20 (83.3)	0.01	35 (62.5)	0.39
Time from transplantation	4.6 ± 2.9	4.3 ± 3.2	0.41	4.2 ± 3.0	0.44	4.2 ± 3.2	0.52	5.1 ± 2.9	0.25
Living donor	98 (45.8)	58 (56.3)	0.08	28 (36.3)	0.21	8 (33.3)	0.24	17 (30.4)	0.04
Donor related	39 (18.4)	31 (30.1)	0.02	14 (18.2)	0.83	3 (12.5)	0.47	14 (25)	0.27
Donor age (y)	41.7 ± 14.8	42.1 ±15.5	0.82	47.6 ±14.4	0.003	47.6 ±14.4	0.06	43.0 ±16.0	0.57
Recipient race									
White	128 (59.8)	69 (67)	0.22	53 (68.9)	0.16	12 (50)	0.36	32 (57.1)	0.71
African-American	52 (24.3)	19 (18.4)	0.24	8 (10.4)	0.01	9 (37.5)	0.16	12 (21.4)	0.65
Hispanics	13 (6.1)	4 (3.9)	0.42	10 (13.0)	0.04	1 (4.2)	0.71	8 (14.3)	0.04
Others	21 (9.8)	11 (10.7)	0.80	6 (7.8)	0.27	2 (8.3)	0.81	1 (1.8)	0.20
Donor sex (male)	90 (42.0)	54 (52.4)	0.08	44 (57.1)	0.02	10 (41.7)	0.97	27 (48.2)	0.41
Diabetes as a cause of ESRD	44 (20.6)	21 (20.4)	0.97	35 (45.5)	<0.001	8 (33.3)	0.15	18 (32.1)	0.07
Dialysis	178 (83.2)	86 (83.5)	0.98	61 (79.2)	0.43	18 (75)	0.32	44 (78.5)	0.41
Prednisone therapy	113 (52.8)	48 (46.6)	0.30	42 (54.5)	0.80	10 (41.7)	0.30	29 (51.8)	0.89
HLA mismatch (A, B, DR)	4.1 ± 1.43	3.8 ± 1.8	0.11	3.5±1.9	0.004	4.5+1.2	0.19	3.6 ± 1.7	0.03
Induction									
Alemtuzumab	79 (36.9)	49 (47.6)	0.07	27 (35.1)	0.78	9 (37.5)	0.95	18 (32.1)	0.51
Thymoglobulin	107 (50.5)	43 (41.2)	0.12	16 (20.8)	<0.001	12 (50)	0.96	29 (51.8)	0.86
Anti-CD25	28 (13.1)	11 (10.7)	0.54	34 (44.2)	<0.001	3 (12.5)	0.93	9 (16.1)	0.56
Clinical factors									
History of any episode of cellular rejection	169 (79.0)	34 (33.6)	<0.001	20 (26)	<0.001	10 (41.6)	<0.001	17 (30.4)	<0.001
History of any episode of acute antibody-mediated rejection	103 (48.6)	7(6.8)	<0.001	1 (1.3)	<0.001	2 (8.3)	<0.001	2 (3.6)	<0.001
History of any episode of chronic antibody-mediated rejection	115 (54.0)	8 (7.8)	<0.001	2 (3.6)	<0.001	1 (4.2)	<0.001	6 (10.7)	<0.001
History of nonskin cancer	13 (6.1)	10 (9.8)	0.24	9 (11.7)	0.11	2(8.3)	0.68	2(3.6)	0.47
History of skin cancer	13 (6.1)	15 (14.7)	0.01	10 (13.0)	0.06	3(12.5)	0.24	10 (17.9)	0.005
History of BK viremia	49 (22.3)	15 (14.7)	0.11	17 (22.7)	0.94	24 (100)	<0.001	9 (16.1)	0.31
History of BK nephropathy	22 (10.3)	6 (5.9)	0.20	4 (5.2)	0.18	24 (100)	<0.001	5 (8.9)	0.76
CMV viremia	25 (11.7)	18 (17.6)	0.15	10(3.0)	0.03	6 (25)	0.07	6 (10.7)	0.83
History of recurrent diarrhea	65 (30.4)	35 (34.3)	0.49	28 (36.4)	0.33	9 (37.5)	0.48	9 (10.6)	0.003
History of nonadherence	85 (39.7)	15 (14.7)	<0.001	9 (11.7)	<0.001	2 (8.3)	0.003	6 (10.7)	<0.001
Immunosuppression reduced by physician	109 (51.2)	43 (42.2)	0.15	39 (50.6)	0.59	24 (100)	<0.001	22 (39.3)	<0.001
Death after graft failed	38 (17.8)	21 (20.4)	0.58	36 (46.7)	<0.001	5 (20.8)	0.125	14 (25)	0.23
De novo DSA	117/197(59.3)	15/90(16.9)	<0.001	5/71 (7)	<0.001	4/18 (22.2)	<0.001	4/45 (7.1)	<0.001
De novo DSA class I only	11 (5.6)	1 (1)	0.07	1 (1.3)	0.14	1 (5.6)	0.95	0 (0)	0.11
De novo DSA class II only	67 (34.0)	10 (9.9)	<0.001	4 (5.3)	<0.001	2 (11.1)	0.04	3 (6.7)	0.002
Both de novo DSA class I and class II	34 (17.3)	4 (4)	0.002	0 (0)	<0.001	1 (5.6)	0.20	1 (2.2)	<0.001
Unknown class DSA (not specified in the chart)	5 (2.5)	0	–	0	–	0	–	0	–

Comparison of each cause of GF compared with alloimmune graft loss reveals different profiles of patient in each category.

BK, polyoma virus; DSA, donor specific antibody; ESRD, end-stage renal disease; GF, graft failure; OR, odds ratio.

Diabetes was more common in patients with tubular injury. Multivariable Cox regression models were developed for specific causes of GF (Tables S10 and S11, SDC, http://links.lww.com/TXD/A398). Note, the comparison is shown but needs to be interpreted in the context of the follow-up period, but these differences are addressed using survival models later.

### Are There Overlapping Risk Factors for DWFG and GF?

**FIGURE 3.**
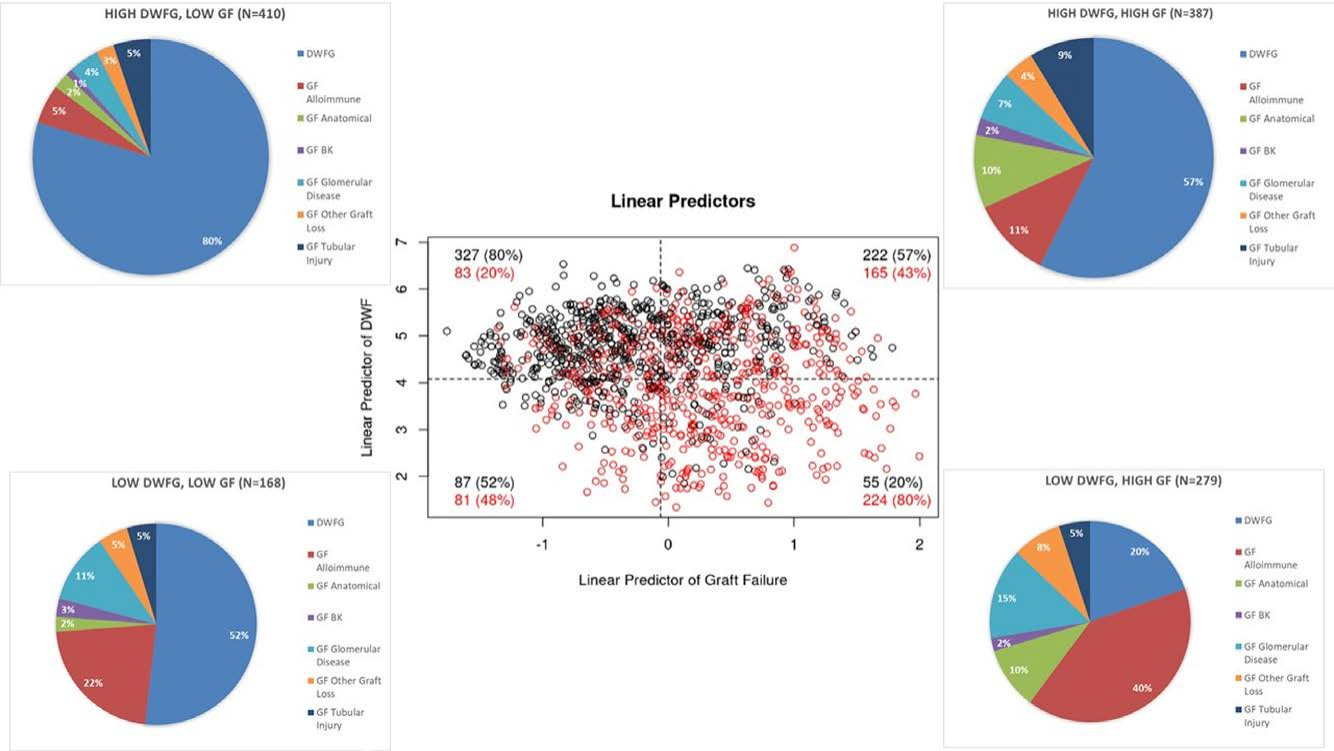
Overlap of risk scores for either DWFG or GF patients at baseline in patients who developed graft loss. In patients who develop either DWFG or GF, we generated risk scores for each outcome based on factors present at the time of transplantation. A, Scatter plot of the linear predictor from cause-specific Cox regression models for GF showing high-risk scores (earlier the overall mean) in many patients. A red circle indicates the patient who actually developed GF, and a black circle indicates the  patient who actually developed DWFG. Each quadrant percentage for GF is displayed to represent the different causes of graft loss. BK, polyoma virus; DWFG, death with a functioning graft; GF, graft failure.

The earlier data suggest that patients with nonalloimmune causes of GF might share similar clinical characteristics at baseline to patients with DWFG. To further explore this, we examined risk scores for DWFG and GF at baseline in all patients who experienced graft loss using a linear predictor model that was normalized around the mean score for the group. Figure 3 shows that 387 of the 1244 patients had high-risk scores for both GF and DWFG at baseline, and the actual cause of graft loss was almost equally split in this group. Higher-risk scores for both GF and DWFG were found in 48.1% of patients with anatomical loss and 44.2% with tubular injury (Figure 3, pie charts). In contrast, in patients with GF due to alloimmunity, only 19.6% had high-risk scores for both.

### Cumulative Incidence Curves Using Competing Risk Analyses

**FIGURE 4.**
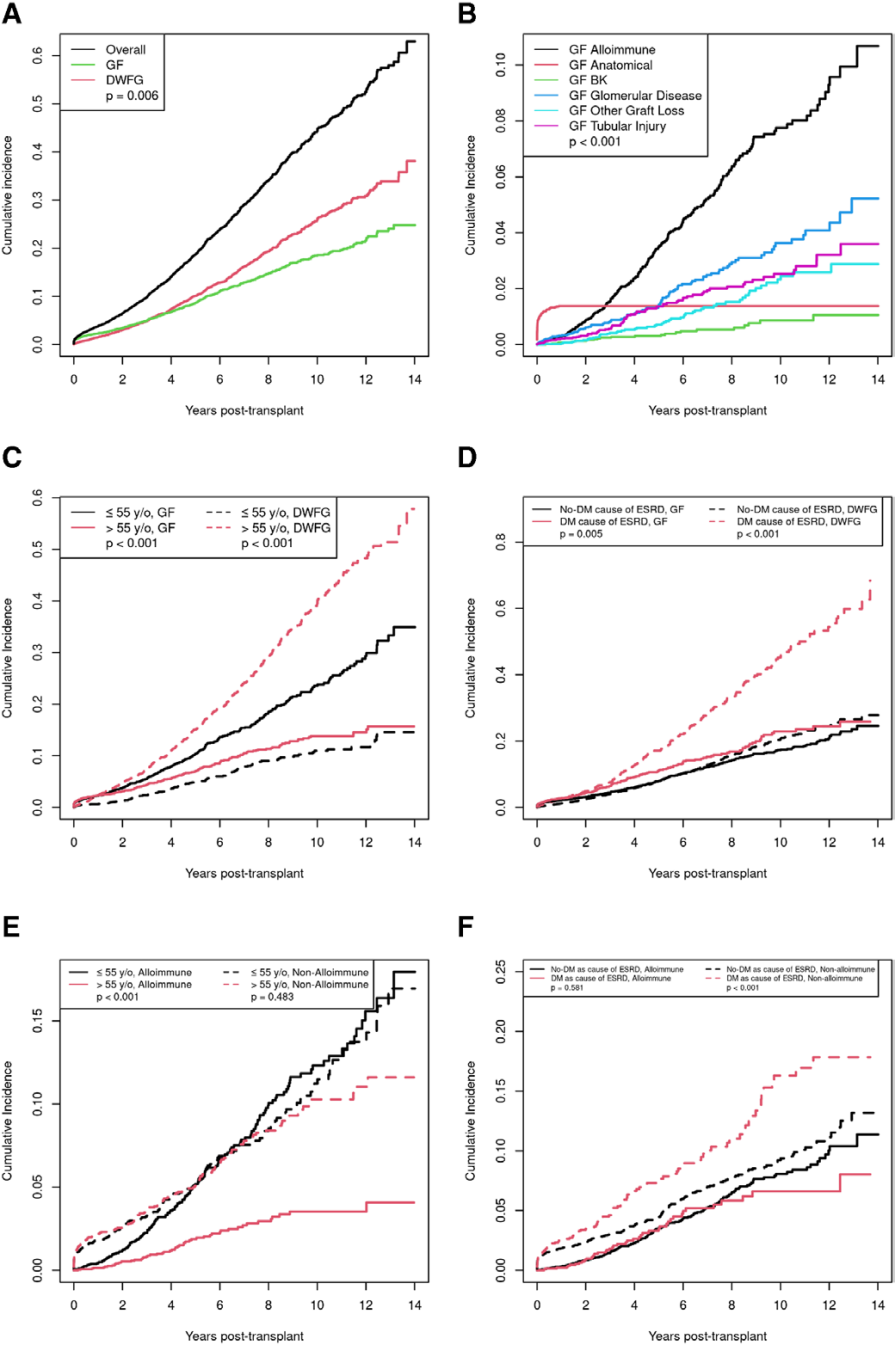
Competing risk cumulative incidences of GF and DWFG. A, Overall graft loss, GF, and DWFG. DWFG is more common over time than GF (*P* = 0.006). B, Incidence curves for each type of GF. Alloimmune causes are more common than all other causes (*P* < 0.001 comparing restricted mean survival). C, GF and DWFG for recipients <55 y old vs >55 y old. GF is higher in younger group (*P* < 0.001), and DWFG is higher in older recipients (*P* < 0.001). D, GF and DWFG in recipients stratified by diabetes as the cause of ESRD. DWFG is more common in diabetics (*P* < 0.001), whereas GF is similar in diabetics and nondiabetics (*P* = 0.071). E, GF due to alloimmune and nonalloimmune causes for recipients <55 vs >55. Younger patients have higher rates of GF due to alloimmune causes (*P* < 0.001); GF due to nonalloimmune causes are similar. F, GF from alloimmune and nonalloimmune causes stratified by diabetes as the cause of ESRD. Diabetics have higher rates of nonalloimmune GF (*P* = 0.002) but similar rates of alloimmune GF. BK, polyoma virus; DWFG, death with a functioning graft; ESRD, end-stage renal disease; GF, graft failure.

Finally, we explored outcomes of patients using survival models adjusted for competing risk. Over time, DWFG was more common than GF (Figure 4A; *P* < 0.001). Figure 4B shows the cumulative incidence of the various causes of GF. Anatomical causes of graft loss occurred early. Other causes of GF increase over time with alloimmune causes higher than the other causes (*P* < 0.001; alloimmune versus the mean of all other causes). Figure 4C compares outcomes in patients ≤55 y old (median age) to those >55 y old. GF was higher in younger recipients (*P* < 0.001), and DWFG was higher in older recipients (*P* < 0.001). Figure 4D shows that patients with diabetes as the cause of ESRD had higher rates of DWFG than those with other causes of ESRD (*P* < 0.001), whereas the relationship was reversed in the case of GF was similar (*P* = 0.005).

Figure 4E compares alloimmune and nonalloimmune GF in 2 age groups. Younger patients (≤55 y old) had higher rates of GF due to alloimmune causes (*P* < 0.001) than patients over 55 y old, whereas GL due to nonalloimmune causes were similar (*P* = 0.483). Figure 4F compares alloimmune and nonalloimmune GL stratified by diabetes as the cause of ESRD. Diabetics have higher rates of nonalloimmune GL (*P* < 0.001) but similar rates of alloimmune GL (*P* = 0.581) compared with nondiabetics.

Taken together, these modeling data suggest there are generally 2 populations of transplant recipients that can be identified at baseline: young, nondiabetic recipients who primarily lose their allograft to alloimmunity and the older, diabetic population at greater risk to lose their graft either to DWFG or to GF (with nonalloimmune causes more likely in this group than in younger, nondiabetic recipients).

## DISCUSSION

The current study is the largest graft loss study from the current era of solid-phase alloantibody testing and tacrolimus-based immunosuppression. Survival models adjusted for competing risk suggested that the rates of DWFG and GF are similar up to 5 y after transplantation, with DWFG becoming more common than GF at 10 y. The incidence of DWFG in our study was similar to a recent era cohort from New Zealand.^6^ In both cohorts, malignancy was the most common cause of DWFG, but in our study, infection ranked higher than cardiac disease. Van Loon et al reported 211 cases of DWFG and, similar to our study, found cancer and infection to be equally common, followed by cardiovascular disease.^7^

In our study, the leading cause of GF was alloimmunity (38.7%), followed by glomerular diseases (18.6%) and renal tubular injury (13.9%). Beyond the first year, alloimmunity accounted for approximately half of the cases of GF. Several recent studies of GF have appeared, and their results have been somewhat variable. Mayrdorfer et al found that alloimmunity accounted for 64.7% of graft losses (T cell–mediated rejection in 34% and AMR in 30.7%).^8^ Medical causes accounted for only 36.3%. Chand et al found that 42.3% of their 97 cases of GF beyond 1 mo were due to alloimmunity.^9^ Interstitial fibrosis/tubular atrophy was a “cause” in 2 studies: van Loon et al (21.4%) and Chand et al (30%).^7,9^ In our own single-center study of GF from the era just before the current study (1996–2006), fibrosis was an attributable cause of graft loss in 31% of cases.^10^ In the current study, we were able to define a specific cause in all but 7.8% of cases and avoided this nonspecific category. Comparing our current study to our prior study, alloimmune causes were higher (39% versus 27%), and glomerular disease was similar (19% versus 21%) (Figure S3, SDC, http://links.lww.com/TXD/A398). Finally, Gaston et al studied 295 cases of GF.^11^ They found that approximately 47.2% were lost because of alloimmunity, and only 2% were due to acute kidney injury.

Gaston et al also examined DWFG and concluded that this population is distinct from those with GF, and our data agreed when we examined the GF population as a whole^11^ ; however, we found important heterogeneity in the GF population. Younger, nondiabetic patients appear to be a distinct population at greater risk to lose their graft to alloimmune-mediated GF. In patients with diabetes as a cause of ESRD that went on to have GF, nonalloimmune causes were more common than alloimmunity. At the time of transplantation, many patients who subsequently developed GF had high-risk scores for both DWFG and GF at baseline. Thus, some patients with GF were more similar at baseline to patients who develop DWFG than they were to other patients who developed GF.

We acknowledge that these different cohorts are heterogeneous. Recurrent renal diseases are common in all age groups, for example; however, we contend that it might be more useful to look at older, diabetic patients as 1 population at baseline who are at risk for both DWFG and GF and to develop management strategies tailored to their needs. Some of the possible approaches include improved management of diabetes, including bariatric surgery; calcineurin-inhibitor free immunosuppression, which has been shown to improve a combined endpoint of patient and graft survival at 7 y^12^; and the avoidance of pretransplant dialysis via preemptive kidney transplantation.^13-15^

Less than 25% of the overall graft losses in our study were due to alloimmunity. Thus, attempts to reduce the incidence of alloimmune-mediated graft loss, although laudable especially for younger patients, are unlikely to significantly change the outcome of the overall population that we are transplanting today. Indeed, it is possible that reduced doses of immunosuppression might actually lead to better outcomes in the older, diabetic cohort.

Our cohort included hundreds of minority recipients; the actual percentage does not replicate the US population (most were White non-Hispanics, and only 13.5% were Black). There was a higher percentage of living donor and preemptive transplants and glomerular disease as a cause of ESRD than the general US kidney transplant population. One of the limitations of our cohort was that the median follow-up was shorter with a median of 3.5 y (interquartile range, 2.0–6.4 y) than other studies and thus may skew the causes of interpretation of GF causes, albeit with 2055 patients with over 5 y of follow-up time from transplant. The lack of available causes of death (n = 256, 37.0%) may reflect sudden cause of death, but statistically, this cohort was not different from the remaining cohort, thus likely reflecting a heterogenous cohort with different causes of death. The strengths of our study included the large number of patients studied with graft loss from the current era, detailed data including protocol biopsies not present in most studies, and our analysis of subgroups. Our use of an adjudication scheme and the use of a category of hemodynamic causes/renal tubular injury were both novel. Few studies of graft loss have included competing risk analyses.^2-4^ Our group has shown that these analyses are important to determine the true risk for GF.

We conclude that new approaches to long-term patient care are needed with greater emphasis on issues related to aging, diabetes, and the complications of immunosuppression. Future clinical trials should be targeting specific causes of graft loss using baseline risk factors to tailor therapy for patient subgroups.

## Supplementary Material



## References

[1] Lamb KE Lodhi S Meier-Kriesche HU. Long-term renal allograft survival in the United States: a critical reappraisal. Am J Transplant. 2011;11:450–462. 20973913 10.1111/j.1600-6143.2010.03283.x

[2] Verduijn M Grootendorst DC Dekker FW. The analysis of competing events like cause-specific mortality–beware of the Kaplan-Meier method. Nephrol Dial Transplant. 2011;26:56–61. 21059831 10.1093/ndt/gfq661

[3] Noordzij M Leffondré K van Stralen KJ. When do we need competing risks methods for survival analysis in nephrology? Nephrol Dial Transplant. 2013;28:2670–2677. 23975843 10.1093/ndt/gft355

[4] El Ters M Smith BH Cosio FG. Competing risk analysis in renal allograft survival: a new perspective to an old problem. Transplantation. 2021;105:668–676. 32332421 10.1097/TP.0000000000003285

[5] Gray RJ. A class of K-sample tests for comparing the cumulative incidence of a competing risk. Ann Stat. 1988;16:1141–1154.

[6] Ying T Shi B Kelly PJ. Death after kidney transplantation: an analysis by era and time post-transplant. J Am Soc Nephrol. 2020;31:2887–2899. 32908001 10.1681/ASN.2020050566 PMC7790214

[7] Van Loon E Senev A Lerut E. Assessing the complex causes of kidney allograft loss. Transplantation. 2020;104:2557–2566. 32091487 10.1097/TP.0000000000003192

[8] Mayrdorfer M Liefeldt L Wu K. Exploring the complexity of death-censored kidney allograft failure. J Am Soc Nephrol. 2021;32:1513–1526. 33883251 10.1681/ASN.2020081215 PMC8259637

[9] Chand S Atkinson D Collins C. The spectrum of renal allograft failure. PLoS One. 2016;11:e0162278. 27649571 10.1371/journal.pone.0162278 PMC5029903

[10] El-Zoghby ZM Stegall MD Lager DJ. Identifying specific causes of kidney allograft loss. Am J Transplant. 2009;9:527–535. 19191769 10.1111/j.1600-6143.2008.02519.x

[11] Gaston RS Fieberg A Helgeson ES; DeKAF Investigators*. Late graft loss after kidney transplantation: is “death with function” really death with a functioning allograft? Transplantation. 2020;104: 1483–1490. 31568212 10.1097/TP.0000000000002961

[12] Vincenti F Rostaing L Grinyo J. Belatacept and long-term outcomes in kidney transplantation. N Engl J Med. 2016;374:333–343. 26816011 10.1056/NEJMoa1506027

[13] Cosio FG Alamir A Yim S. Patient survival after renal transplantation: I. The impact of dialysis pre-transplant. Kidney Int. 1998;53:767–772. 9507225 10.1046/j.1523-1755.1998.00787.x

[14] Meier-Kriesche HU Kaplan B. Waiting time on dialysis as the strongest modifiable risk factor for renal transplant outcomes: a paired donor kidney analysis. Transplantation. 2002;74:1377–1381. 12451234 10.1097/00007890-200211270-00005

[15] Innocenti GR Wadei HM Prieto M. Preemptive living donor kidney transplantation: do the benefits extend to all recipients? Transplantation. 2007;83:144–149. 17264810 10.1097/01.tp.0000250555.46539.65

