# Supplementary material for: Death With Function and Graft Failure After Kidney Transplantation: Risk Factors at Baseline Suggest New Approaches to Management
Source: Transplant Direct. 2022 Jan 13;8(2):e1273. doi: 10.1097/TXD.0000000000001273 (PMC8759617; doi:10.1097/TXD.0000000000001273)
Supplement: Supplementary file 1 [file txd-8-e1273-s001.pdf]

Supplementary Data. Causes of Kidney Allograft Loss

Table of Content

Supplementary Methods ……………………………………………………………………………………………………2-9
Table S1 Timeline of graft loss, death with functioning graft …………10

Table S2 Multivariable Analysis of cardiac causes for death with functioning graft...11

Table S3. Multivariable Analysis for malignancy as a cause for death with functioning graft…….………………12

Table S4. Multivariable Analysis for infection as a cause for death with functioning graft……….......13

Table S5. Assignment and agreement of causes of graft loss between physicians…....14
Table S6 Specific causes of death censored graft loss…………………...…….…………….15 -16

Table S7 Multivariable analysis for all Death Censored Graft.…………………………………17
Table S8 Multivariable analysis for Alloimmune Causes ……………………………………..…..18

Table S9. Multivariable analysis for Renal Tubular Injuries Causes…………………………19

Table S10 Multivariable Analysis for overall death with functioning graft…………….…20

Table S11. Baseline Characteristic comparison between the ones with known cause of death and unknown cause of death ………………………………………………………………………………….……21

Figure S1. Flowchart study population...……………………………………………………….....22

Figure S2 Causes of Death Censored Graft Loss during time……………………………………….23

Figure S3. Comparison of Causes of Allograft Failure in Two Eras……………..............24

Supplementary Data.

1. **Supplementary Methods**

Follow up protocol

Patients in this study were followed using a common protocol that included both surveillance biopsies at 4-8 months, 1 year, 2 years, 4-5 year and 8-10 years and biopsies for cause. Patient also had laboratory follow up: in the first year weekly early than monthly, after one-year laboratory tests were obtained every 3-6 months. Initial maintenance regimen was primarily tacrolimus, mycophenolate mofetil (MMF), +/- prednisone.

2. **Determining the cause of GF.**

A. Definitions of the causes of graft loss

1) Alloimmune causes of graft losses were classified by using Banff Classification 2017 criteria for acute active antibody mediated rejection and chronic active mediated rejection. Cellular rejection was determine by having acute cellular rejection or borderline rejection or chronic T cell rejection in the absence of antibody mediated injury.

a) T cell Rejection was defined by having Acute Cellular Rejection or chronic active cellular rejection: It was defined by acute cellular rejection or chronic cellular rejection or borderline cellular rejection in biopsy or documentation in the electronic medical chart in the absence of antibody mediated injury or documentation of it.

b) Acute Active Antibody-Mediated Rejection was defined as acute active antibody mediated injury as per Banff 2017 in the absence of cellular rejection or documentation of it.

c) Mixed Acute Cellular Rejection and Active Antibody-Mediated Rejection: It was defined as having a combination of a) and b) in biopsy or documentation of it.

d) Chronic Active Antibody-Mediated Rejection was defined as having chronic active antibody mediated rejection by Banff 2017 or documentation of transplant glomerulopathy or chronic antibody mediated rejection but in the absence any cellular rejection component in last biopsy.

e) Chronic Active Antibody-Mediated Rejection with cellular component: It was defined as having a combination of a) and d) in biopsy or documentation of it definitions or documentation of it.

2) Glomerular Cause were assigned etiologies that are non-alloimmune that affect the glomeruli.

a) Recurrence glomerulonephritis was defined as a recurrent glomerular disease same as native that leads to allograft loss.

b) De novo glomerular diseases were classified as having new onset glomerular disease after transplantation that leads to graft loss.

c) Diabetic nephropathy was defined as diabetic nephropathy as the cause of renal allograft loss.

d) Thrombotic microangiopathy defined as the pathological lesion thrombotic microangiopathy that leads to graft loss that is not associated with alloimmune injury.

3) Renal Tubular Injury Causes were attributed to systemic illness leading to either acute or repeated acute kidney injury which progressed to allograft failure, for example sepsis with hypotension and non-recovered kidney function, repeat urinary tract infections without other cause of allograft injury determined by clinical that were accompanied with or without histology

a) An episode of acute tubular necrosis: It was defined when one episode with acute tubular necrosis lead to graft loss.

b) Recurrent episode of acute tubular necrosis due to infection: It was defined as having recurrent episodes of acute kidney injury due to infection

c) Recurrent episode of acute tubular necrosis due to hypovolemia: It was defined as having recurrent episodes of acute kidney injury due to infection

d) Nephrotoxic Medication was defined as any tubular injury caused by other medication that is not a calcineurin inhibitor.

e) Calcineurin inhibitor toxicity= renal failure due to calcineurin inhibitor without a second insult.

f) Oxalate nephropathy= Allograft failure due to oxalate nephropathy

g) Other recurrent episodes acute tubular necrosis: Recurrent episodes of acute tubular necrosis.

h) Secondary to cardiorenal syndrome: Renal failure due to cardiorenal syndrome causing renal allograft failure.

i) Secondary to hepatorenal syndrome: Renal failure due to hepatorenal syndrome causing renal allograft failure.

4) Surgical complication defined as grafts that never had a good function or either had a technical complication that lead to graft failure in the first year.

a) Vascular Thrombosis= Graft loss causes either by arterial or vein thrombosis of the renal allograft

b) Bleeding: Graft loss caused by bleeding

c) Urological complication: Any surgical complication that involves the ureter or bladder

d) Other complication secondary to infection: Surgical complication that lead infection leading to graft loss

e) Vascular atherosclerosis: Atherosclerosis of the artery that lead to graft loss the first year

f) Primary Dysfunction was defined as a graft that never had any function

5) BK nephropathy= was defined as the cause that had biopsy proven BK nephropathy that either was active (in the last biopsy) or inactive (negative in the last biopsy) but had chronic lesions.

a) Active BK nephropathy= This is due to active lesion of BK nephropathy

b) Inactive BK nephropathy= This is due chronic lesions BK nephropathy but not active in the last biopsy.

6) Others: Other causes were assigned in the different subcategories that were unable to group.

a) Unknown causes was assigned to causes that were lack of sufficient clinical, laboratory data or evidence for graft loss.

b) Atherosclerosis vascular disease: are the causes due to atherosclerosis of the vessels that cause graft loss after 1 year

c) Suboptimal kidney function that survive greater than 1 year: Kidney allograft that never had a good renal function but that failed after one year of transplant.

d) Severe Arteriolar hyalinosis (without any other cause) was defined as arteriolar hyalinosis of the allograft that is not associated with other cause.

e) Malignancy in the allograft= It was defined as a malignancy in the renal or urothelial of the allograft that lead to nephrectomy.

B. Causes of Definition of Death with functioning graft

The causes of death with functioning allograft were determined by a transplant nephrologist that did electronic medical chart review of the causes of death with functioning. They were classified in the different categories

a) Cardiac: Defined as the cause of death due to a cardiac cause.

b) Malignancy: Defined as the cause of death due to malignancy.

c) Infection: Defined as the cause of death due to infection.

d) Unknown: Defined as death without unknown cause either due to lack of information or documentation to reach to the cause.

e) Others: All other causes that are not applicable of prior causes of death. Between the other causes were stroke, trauma, bleeding, pulmonary causes, neurological causes, pancreatitis, liver failure or complications renal failure that went for palliative care option without any renal replacement therapy.

C. Definitions of the variables

These are the definitions of our variables:

1) Diabetes Mellitus as a cause of pretransplant ESRD was defined by documentation diagnosis as diabetes mellitus as the cause of native kidney disease.
2) Delayed Graft Functioning was defined as any dialysis required in the first week after transplantation obtained from the UNOS STAR data.
3) Calculated Panel Reactive Antigen score (cPRA) was obtained from UNOS STAR data reported pretransplant.

Clinical Characteristics of the variables from death censored graft loss

4) History of any episode of T cell mediated rejection= Any T cell rejection by Banff 2017 including borderline cellular rejection or chronic active T cell mediated rejection or description from outside study. This data was obtained by data abstraction for only the patients with death censored graft loss.
5) History of any episode of acute antibody mediated rejection= Antibody mediated rejection by Banff Classification 2017 during the transplant period regardless if the biopsy has transplant glomerulopathy (cg) or description from outside study. This data was obtain by data abstraction for only the patients with death censored graft loss.
6) History of any episode of Chronic Antibody Mediated rejection = any transplant glomerulopathy (cg) in kidney biopsy during the transplant period or description from outside study. This data was obtained by data abstraction for only the patients with death censored graft loss.

7) History of Non-skin cancer= Any malignancy other from skin source during the transplant period. This data was obtain by data abstraction for only the patients with death censored graft loss.

8) BK viremia was defined as having any serum BK or documentation of it during the transplant period. This data was obtain by data abstraction for only the patients with death censored graft loss.

9) CMV viremia was defined as having any serum CMV or documentation of it during the transplant period. This data was obtain by data abstraction for only the patients with death censored graft loss.

10) History of recurrent/chronic diarrhea= Any diarrhea that has 4 weeks of diarrhea in a year or having equal or greater than 3 episodes of diarrhea during a year. This data was obtain by data abstraction for only the patients with death censored graft loss.

11) Noncompliance = Description or documentation referring that the patient is noncompliant, no adherent, missing doses or skipping doses of immunosuppression in the medication This data was obtain by data abstraction for only the patients with death censored graft loss.

12) Immunosuppression decreased by provider was defined as:

a. Reduction of mycophenolic mofetil or mycophenolic acid (MPA) dose lower than <500mg PO BID or 360 mg PO BID or holding/discontinuing the antimetabolite for any cause. (Example BK nephropathy, malignancy)

b. Decrease in calcineurin inhibitor target through goal level (cyclosporine or tacrolimus target through level) or discontinuation without substitution.

c. Change of mycophenolic mofetil or mycophenolic acid to azathioprine (AZT).

13) De novo Donor Specific Antibodies= New donor specific antibodies that are donor specific antibodies with an MFI greater than 1000 after transplantation or documentation from outside facility that the patient had de novo DSA.

Table S1 Timeline Of Graft Loss And Death With Functioning Graft

Status of the transplant	Up to 1 year follow up	Up to 5 years follow up	Greater than 5 years follow up	Overall
DWFG	85 (1.5%)	399 (6.9%)	647 (11.25%)	691 (12.0%)
Graft failed	131 (2.3%)	365 (6.3%)	527 (9.16%)	553 (9.6%)
Active (Last follow up <1 year from censor date)	53 (93.8%)	1605 (64.6%)	1150 (50.85%)	2808(48.8%)
Active (Last follow up ≥1 year from censor date)	140 (2.4%)	1132 (22.1%)	428 (28.74%)	1700 (29.6%)

Table S2 Multivariable Analysis of cardiac causes for death with functioning graft

Cardiac Cause of death	Unadjusted			Adjusted*		
Clinical Characteristics	HR	95% CI	P-value	HR	95% CI	P-value
Age, 10 years	1.68	1.38-2.03	<0.001	1.57	1.28-1.92	<0.001
Sex (Male)	1.65	1.04-2.63	0.034	1.43	0.89-2.28	0.135
Deceased Donor	1.19	0.78-1.82	0.428	-	-	-
Period 2006-2009	1.00	Ref	Ref	1.00	Ref	Ref
Period 2010-2013	0.66	0.39-1.12	0.125	-	-	-
Period 2014-2018	0.79	0.40-1.56	0.502	-	-	-
Prior Kidney Transplant	0.79	0.36-1.70	0.541	-	-	-
Dialysis	0.97	0.63-1.50	0.895	-	-	-
Prednisone Therapy	1.12	0.73-1.71	0.615	-	-	-
Induction (thymoglobulin and antiCD25) vs. (Alemtuzumab)	1.45	0.87-2.41	0.151	-	-	-
Donor age, 10 years	1.04	0.89-1.21	0.647	-	-	-
Donor Gender	0.82	0.54-1.25	0.355	-	-	-
HLA number Mismatch (A, B, DR)	0.10	0.98-1.26	0.116	-	-	-
Donor Black race	0.79	0.32-1.96	0.614	-	-	-
Recipient Black race	1.17	0.64-2.16	0.603	-	-	-
Diabetes Mellitus as Cause of ESRD	3.58	2.34-5.49	<0.001	2.69	1.74-4.18	<0.001
Delayed Graft Function	1.76	1.05-2.94	0.031	1.44	0.85-2.42	0.174
HLA A, B mismatch	1.10	0.93-1.30	0.267	-	-	-
HLA DR Mismatch	1.34	0.99-1.81	0.061	-	-	-
CPRA≥80% (only in 4941)	1.35	0.58-3.15	0.492	-	-	-

ESRD: End Stage Renal Disease; DGF: Delayed Graft Function; CPRA: Calculated Panel Reactive Antibody; HLA: Human Leukocyte Antigen.

* Adjusted for recipient age, sex, delayed graft functioning, diabetes mellitus as a cause pre-transplant of ESRD

Table S3. Multivariable Analysis for malignancy as a cause for death with functioning graft

Malignancy As Cause Of Death	Unadjusted			Adjusted*		
Clinical Characteristics	HR	95%CI	P-value	HR	95%CI	P-value
Age, 10 years	1.86	1.58-2.18	<0.001	1.79	1.51-2.12	<0.001
Sex (Male)	2.04	1.38-3	<0.001	1.81	1.22-2.67	0.003
Deceased Donor	1.41	1.01-1.97	0.046	1.05	0.73-1.51	0.809
Period 2006-2009	1.00	Ref	Ref	1.00	Ref	Ref
Period 2010-2013	1.53	1.02-2.3	0.04	1.49	0.99-2.23	0.056
Period 2014-2018	1.66	0.97-2.83	0.062	1.61	0.94-2.76	0.082
Prior Kidney Transplant	0.94	0.53-1.66	0.828	-	-	-
Dialysis	1.62	1.11-2.37	0.012	1.64	1.1-2.44	0.015
Prednisone Therapy	1.26	0.9-1.78	0.181	-	-	-
Induction (thymoglobulin and antiCD25) vs. (Alemtuzumab)	1.29	0.87-1.9	0.202	-	-	-
Donor age, 10 years	1.16	1.03-1.31	0.018	1.05	0.93-1.18	0.473
Donor Gender	0.95	0.68-1.32	0.753	-	-	-
HLA number Mismatch (A, B, DR)	0.99	0.9-1.09	0.83	-	-	-
Donor Black race	0.60	0.26-1.35	0.214	-	-	-
Recipient Black race	0.96	0.57-1.62	0.88	-	-	-
Diabetes Mellitus as Cause of ESRD	1.81	1.25-2.61	0.002	1.28	0.88-1.86	0.19
Delayed Graft Function	1.06	0.66-1.71	0.801	-	-	-
HLA A, B mismatch	1.03	0.9-1.17	0.686	-	-	-
HLA DR Mismatch	0.86	0.68-1.09	0.208	-	-	-
CPRA≥80% (only in 4941)	0.77	0.36-1.66	0.507	-	-	-

ESRD: End Stage Renal Disease; DGF: Delayed Graft Function; CPRA: Calculated Panel Reactive Antibody; HLA: Human Leukocyte Antigen.
* Adjusted for recipient age, sex, deceased donor, period of transplant, donor ager and diabetes mellitus as a pre-transplant cause of ESRD

Table S4. Multivariable Analysis for infection as a cause for death with functioning graft

Infection As Cause Of Death	Unadjusted			Adjusted*		
Clinical Characteristics	HR	95%CI	P-value	HR	95%CI	P-value
Age, 10 years	2.14	1.8-2.54	<0.001	1.93	1.59-2.33	<0.001
Sex (Male)	1.26	0.88-1.79	0.207			
Deceased Donor	2.08	1.47-2.94	<0.001	1.46	0.95-2.24	0.087
Period 2006-2009	1.00	Ref	Ref	1.00	Ref	Ref
Period 2010-2013	1.12	0.73-1.72	0.607	-	-	-
Period 2014-2018	1.28	0.79-2.09	0.316	-	-	-
Prior Kidney Transplant	0.94	0.53-1.67	0.842	-	-	-
Dialysis	1.88	1.25-2.8	0.002	1.75	1.14-2.68	0.011
Prednisone Therapy	1.92	1.33-2.78	<0.001	1.81	1.13-2.91	0.013
Induction (thymoglobulin and antiCD25) vs. (Alemtuzumab)	1.87	1.23-2.84	0.003	0.83	0.49-1.4	0.491
Donor age, 10 years	1.27	1.12-1.44	<0.001	1.12	0.99-1.27	0.065
Donor Gender	1	0.72-1.41	0.977	-	-	-
HLA number Mismatch (A, B, DR)	1.13	1.01-1.25	0.026	1.07	0.82-1.41	0.606
Donor Black race	0.79	0.38-1.61	0.508	-	-	-
Recipient Black race	0.88	0.51-1.5	0.634	-	-	-
Diabetes Mellitus as Cause of ESRD	2.19	1.53-3.12	<0.001	1.55	1.07-2.23	0.02
Delayed Graft Function	1.55	1.03-2.33	0.037	0.93	0.59-1.46	0.745
HLA A, B mismatch	1.16	1.01-1.33	0.039	-	-	-
HLA DR Mismatch	1.24	0.97-1.58	0.081	-	-	-
CPRA≥80% (only in 4941)	1.09	0.57-2.1	0.792	-	-	-

ESRD: End Stage Renal Disease; DGF: Delayed Graft Function; CPRA: Calculated Panel Reactive Antibody; HLA: Human Leukocyte Antigen.

* Adjusted for recipient age, deceased donor, dialysis pre-transplant, prednisone, induction, HLA mismatch, donor age, Delayed Graft Function and diabetes mellitus as a pre-transplant cause of ESRD

Table S5. Assignment and agreement of causes of graft loss between physicians.

	Total Assigned	First Assigned Cause Agreement Between MD1 and MD2)	First Disagreement	Assigned Cause by 3rd MD	Assigned Cause by 4th MD
Alloimmune	214	186	28	19	9
Glomerular	103	92	11	8	3
Renal Tubular	77	56	21	17	4
BK	24	21	3	1	2
Anatomic	79	71	8	8	0
Other	56	36	20	16	4
Total	553	462	91	69	22

Table S6. Specific causes of graft losses

Causes of allograft loss	N	Categories
1. Alloimmune= Cause of failure due to rejection	214	
1.1 Acute Cellular Rejection or chronic active cellular rejection	44	20.6%
1.2 Acute Active Antibody-Mediated Rejection	2	0.9%
1.3 Mixed Acute Cellular Rejection and Active Antibody-Mediated Rejection	55	25.7%
1.4 Chronic Active Antibody-Mediated Rejection	71	33.2%
1.5 Chronic Active Antibody-Mediated Rejection and Acute Cellular Rejection	42	19.6%
2. Glomerular Disease	103	
2.1 Recurrent Glomerulonephritis	61	59.2%
2.2 De novo Glomerulonephritis	10	9.7%
2.3 Diabetic Nephropathy without any other cause	15	14.6%
2.4 Thrombotic Microangiopathy	17	16.5%
3. Renal Tubular Injury	77	
3.1 One episode of Acute Tubular Necrosis	13	16.9%
3.2 Recurrent episodes of Acute Tubular Necrosis due to infection	30	39.0%
3.3 Recurrent episodes of Acute Tubular Necrosis due to hypovolemia	11	14.3%
3.4 Nephrotoxic medication (other than CNI e.g. Amphotericin B, contrast nephropathy)	1	1.3%
3.5 Calcineurin inhibitor toxicity that does not have a second insult	0	0.0%
3.6 Oxalate Nephropathy	3	3.9%
3.7 Other recurrent episodes of Acute Tubular Necrosis	5	6.5%
3.8 Secondary to Cardiorenal Syndrome	12	15.6%
3.9 Secondary to Hepatorenal Syndrome	2	2.6%
4.0 Surgical/Anatomical complication	79	
4.1 Vascular thrombosis	45	57.0%
4.2 Bleeding	4	5.1%
4.3 Urological complication	0	0.0%
4.4 Other complication secondary to infection	1	1.3%
4.5 Vascular Atherosclerosis	1	1.3%
4.6 Primary Dysfunction	28	35.4%

5. BK nephropathy	24	
5.1 Active BK nephropathy	12	50.0%
5.2 Secondary to BK nephropathy not active in last biopsy	12	50.0%
6. Other	56	
6.1 CMV nephropathy	0	0.0%
6.2 Severe Arteriolar Hyalinosis (without other cause)	3	5.4%
6.3 Adenovirus nephritis	1	1.8%
6.4 Unknown	39	69.6%
6.5 Dihydroxyadenine crystalline nephropathy	1	1.8%
6.6 Malignancy in the renal allograft (PTLD, urothelial carcinoma…)	2	3.6%
6.7 Nephroptosis with Vascular torsion	1	1.8%
6.8 Atherosclerosis vascular disease	4	7.1%
6.9 Suboptimal kidney function that survive greater than 1 year	4	7.1%
6.10 Cryptococcus infection of the kidney	1	1.8%

BK: Polyoma Virus
CMV: Cytomegalovirus
PTLD: Post-Transplant Lymphoproliferative Disease

Table S7. Multivariable analysis for all Graft Loss

Death Censored graft loss	Unadjusted			Adjusted*		
Clinical Characteristics	HR	95%CI	P-value	HR	95%CI	P-value
Age, 10 years	0.84	0.79-0.89	<0.001	0.8	0.75-0.85	<0.001
Sex (Male)	1.11	0.94-1.32	0.227	-	-	-
Deceased Donor	1.9	1.6-2.25	<0.001	1.16	0.93-1.45	0.198
Period 2006-2009	1	Ref	Ref	1	Ref	Ref
Period 2010-2013	0.97	0.79-1.19	0.758	-	-	-
Period 2014-2018	1	0.78-1.27	0.972	-	-	-
HLA number Mismatch (A, B, DR)	1.11	1.05-1.16	<0.001	*	*	*
Prior Kidney Transplant	1.32	1.03-1.69	0.031	1.33	1.01-1.74	0.042
Dialysis	2.39	1.93-2.95	<0.001	1.54	1.22-1.95	<0.001
Prednisone Therapy	0.85	0.72-1	0.048	1.02	0.82-1.26	0.88
Induction (thymoglobulin and antiCD25) vs. (Alemtuzumab)	0.83	0.69-0.98	0.033	1.05	0.84-1.3	0.671
Donor age, 10 years	1.04	0.98-1.1	0.216	-	-	-
Donor Gender	0.92	0.78-1.08	0.308	-	-	-
Donor Black Race	1.65	1.27-2.14	<0.001	1.35	1.02-1.78	0.038
Recipient Black Race	1.9	1.55-2.33	<0.001	1.4	1.1-1.79	0.006
Diabetes Mellitus as Cause of ESRD	1.48	1.23-1.79	<0.001	1.4	1.14-1.72	0.002
Delayed Graft Function	3.18	2.67-3.8	<0.001	2.2	1.78-2.73	<0.001
HLA A, B mismatch	1.11	1.04-1.19	0.002	0.97	0.89-1.05	0.431
HLA DR Mismatch	1.27	1.13-1.43	<0.001	1.27	1.11-1.45	<0.001
CPRA≥80% (only in 4941)	1.32	0.97-1.78	0.078	-	-	-

ESRD: End Stage Renal Disease; DGF: Delayed Graft Function; CPRA: Calculated Panel Reactive Antibody; HLA: Human Leukocyte Antigen.

* Adjusted for age, deceased donor, HLA A,B mismatch, HLA DR mismatch, induction, dialysis pre-transplant, prednisone therapy, prior kidney transplant, donor race, recipient race, delayed graft functioning, diabetes mellitus as a cause pre-transplant of ESRD and transplant site

Table S8. Multivariable analysis for Alloimmune Causes

Alloimmune causes	Unadjusted			Adjusted*		
Clinical Characteristics	HR	95%CI	P-value	HR	95%CI	P-value
Age, 10 years	0.64	0.58-0.7	<0.001	0.64	0.57-0.71	<0.001
Sex (Male)	0.86	0.66-1.13	0.288	-	-	-
Deceased Donor	1.68	1.28-2.2	<0.001	1.6	1.05-2.44	0.029
Period 2006-2009	1.00	Ref	Ref	1.00	Ref	Ref
Period 2010-2013	1.04	0.75-1.42	0.829	-	-	-
Period 2014-2018	1	0.66-1.52	0.984	-	-	-
HLA number Mismatch (A, B, DR)	1.21	1.11-1.32	<0.0001	-	-	-
Prior Kidney Transplant	1.58	1.08-2.3	0.017	1.03	0.63-1.69	0.914
Dialysis	2.89	2.02-4.14	<0.001	2.15	1.34-3.46	0.001
Prednisone Therapy	0.89	0.68-1.16	0.374	-	-	-
Induction (thymoglobulin and antiCD25) vs. (Alemtuzumab)	0.76	0.57-1.01	0.06	-	-	-
Donor age, 10 years	0.95	0.86-1.04	0.257	-	-	-
Donor Gender	1.68	1.28-2.2	<0.001	1.60	1.05-2.44	0.029
Donor Black race	1.03	0.62-1.72	0.9	-	-	-
Recipient Black race	2.31	1.69-3.16	<0.001	1.79	1.19-2.7	0.005
Diabetes Mellitus as Cause of ESRD	1.08	0.78-1.51	0.643	-	-	-
Delayed Graft Function	2.05	1.5-2.81	<0.001	1.27	0.85-1.91	0.251
HLA A, B mismatch	1.22	1.09-1.36	<0.001	1.00	0.83-1.16	0.963
HLA DR Mismatch	1.6	1.31-1.95	<0.001	1.56	1.21-2.01	0.0006
CPRA≥80 (only in 4941)	2.61	1.75-3.87	<0.001	2.19	1.37-3.48	<0.001

ESRD: End Stage Renal Disease; DGF: Delayed Graft Function; CPRA: Calculated Panel Reactive Antibody; HLA: Human Leukocyte Antigen.
* Adjusted for age, deceased donor, HLA A, B mismatch, HLA DR mismatch, dialysis pre-transplant, prior kidney transplant, recipient race, delayed graft functioning, diabetes mellitus as a cause pre-transplant of ESRD, CPRA≥80%

Table S9. Multivariable analysis for Renal Tubular Injuries Causes

Renal Tubular injuries	Unadjusted			Adjusted*		
Clinical Characteristics	HR	95%CI	P-value	HR	95%CI	P-value
Age, 10 years	1.32	1.1-1.58	0.003	1.10	0.91-1.37	0.295
Sex (Male)	1.04	0.66-1.65	0.863	-	-	-
Deceased Donor	2.41	1.51-3.85	<0.001	1.75	0.94-3.25	0.075
Period 2006-2009	1.00	Ref	Ref	1.00	Ref	Ref
Period 2010-2013	0.92	0.51-1.66	0.784	-	-	-
Period 2014-2018	1.8	0.96-3.38	0.066	-	-	-
Prior Kidney Transplant	1.04	0.5-2.16	0.916	-	-	-
Dialysis	2.17	1.25-3.77	0.006	1.44	0.78-2.66	0.247
Prednisone Therapy	0.96	0.61-1.5	0.841	-	-	-
Induction (thymoglobulin and antiCD25) vs. (Alemtuzumab)	0.86	0.54-1.39	0.549	-	-	-
Donor age, 10 years	1.33	1.12-1.57	0.001	1.27	1.07-1.5	0.006
Donor Gender	1.34	0.86-2.11	0.198	-	-	-
HLA number Mismatch (A, B, DR)	0.97	0.86-1.11	0.686	-	-	-
Donor Black race	1.45	0.7-3.02	0.316	-	-	-
Recipient Black race	0.82	0.4-1.72	0.606	-	-	-
Diabetes Mellitus as Cause of ESRD	3.48	2.21-5.47	<0.001	2.41	1.49-3.9	<0.001
Delayed Graft Function	4.27	2.7-6.76	<0.001	2.72	1.56-4.76	<0.001
HLA A, B mismatch	0.96	0.81-1.14	0.649	-	-	-
HLA DR Mismatch	0.97	0.71-1.33	0.863	-	-	-
CPRA≥80% (only in 4941)	0.41	0.1-1.67	0.211	-	-	-

ESRD: End Stage Renal Disease; DGF: Delayed Graft Function; CPRA: Calculated Panel Reactive Antibody; HLA: Human Leukocyte Antigen.

* Adjusted for age, deceased donor, dialysis pre-transplant, donor age, delayed graft functioning,

diabetes mellitus as a cause pre-transplant of ESRD.

Table S10. Multivariable Analysis for overall Death with Functioning Graft

Death with functioning graft	Unadjusted			Adjusted*		
Clinical Characteristics	HR	95%CI	P-value	HR	95%CI	P-value
Age, 10 years	1.91	1.78-2.05	<0.001	1.79	1.66-1.95	<0.001
Sex (Male)	1.53	1.3-1.8	<0.001	1.34	1.14-1.58	<0.001
Deceased Donor	1.73	1.48-2.01	<0.001	1.13	0.94-1.37	0.197
Period 2006-2009	1	Ref	Ref	1	Ref	Ref
Period 2010-2013	1.18	0.98-1.42	0.08	1.04	0.85-1.28	0.696
Period 2014-2018	1.48	1.18-1.87	<0.001	1.23	0.96-1.59	0.102
Prior Kidney Transplant	0.83	0.63-1.08	0.163	-	-	-
Dialysis Pretransplant	1.65	1.39-1.95	<0.001	1.49	1.24-1.78	<0.001
Prednisone Therapy	1.32	1.13-1.54	<0.001	1.34	1.08-1.67	0.008
Induction (thymoglobulin and antiCD25)vs (Alemtuzumab)	1.46	1.22-1.75	<0.001	0.91	0.72-1.15	0.421
Donor age, 10 years	1.12	1.06-1.18	<0.001	1.02	0.97-1.07	0.50
Donor Gender	1.02	0.88-1.19	0.783	-	-	-
HLA number Mismatch (A, B, DR)	1.02	0.97-1.06	0.462	-	-	-
Donor African American	1.03	0.77-1.37	0.836	-	-	-
Recipient African American	0.92	0.73-1.17	0.511	-	-	-
Diabetes Mellitus	2.65	2.27-3.09	<0.001	1.88	1.6-2.21	<0.001
DGF	1.65	1.38-1.99	<0.001	1.1	0.89-1.36	0.365
HLA AB Mismatch	1.02	0.96-1.08	0.45	-	-	-
HLA DR Mismatch	1.02	0.92-1.14	0.66	-	-	-
CPRA≥80 (only in 4941)	1	0.73-1.36	0.976	-	-	-

ESRD: End Stage Renal Disease; DGF: Delayed Graft Function; CPRA: Calculated Panel Reactive Antibody; HLA: Human Leukocyte Antigen.

* Adjusted for recipient age, sex, deceased donor, period of transplant, induction, dialysis pretransplant, prednisone therapy, delayed graft functioning, diabetes mellitus as a cause pretransplant of ESRD and transplant site.

Table S11. Baseline Characteristic comparison between the ones with known cause of death and unknown cause of death

	Known COD (N=435)	Unknown COD (N=256)	Total (N=691)	p value
**Age per 10 years**				0.559
Mean (SD)	6.247 (1.009)	6.295 (1.074)	6.265 (1.033)	
Range	2.700 - 8.200	2.300 - 8.400	2.300 - 8.400	
**Donor Type**				0.073
Mean (SD)	0.520 (0.500)	0.590 (0.493)	0.546 (0.498)	
Range	0.000 - 1.000	0.000 - 1.000	0.000 - 1.000	
**Previous Kidney Transplants**				0.480
Mean (SD)	0.090 (0.286)	0.074 (0.263)	0.084 (0.277)	
Range	0.000 - 1.000	0.000 - 1.000	0.000 - 1.000	
**pretransplant dialysis**				0.251
Mean (SD)	0.722 (0.449)	0.762 (0.427)	0.737 (0.441)	
Range	0.000 - 1.000	0.000 - 1.000	0.000 - 1.000	
**Steroid Maintenance (1)**				0.141
Mean (SD)	0.646 (0.479)	0.590 (0.493)	0.625 (0.484)	
Range	0.000 - 1.000	0.000 - 1.000	0.000 - 1.000	

<u>Figure S1. Flowchart of the study population</u>

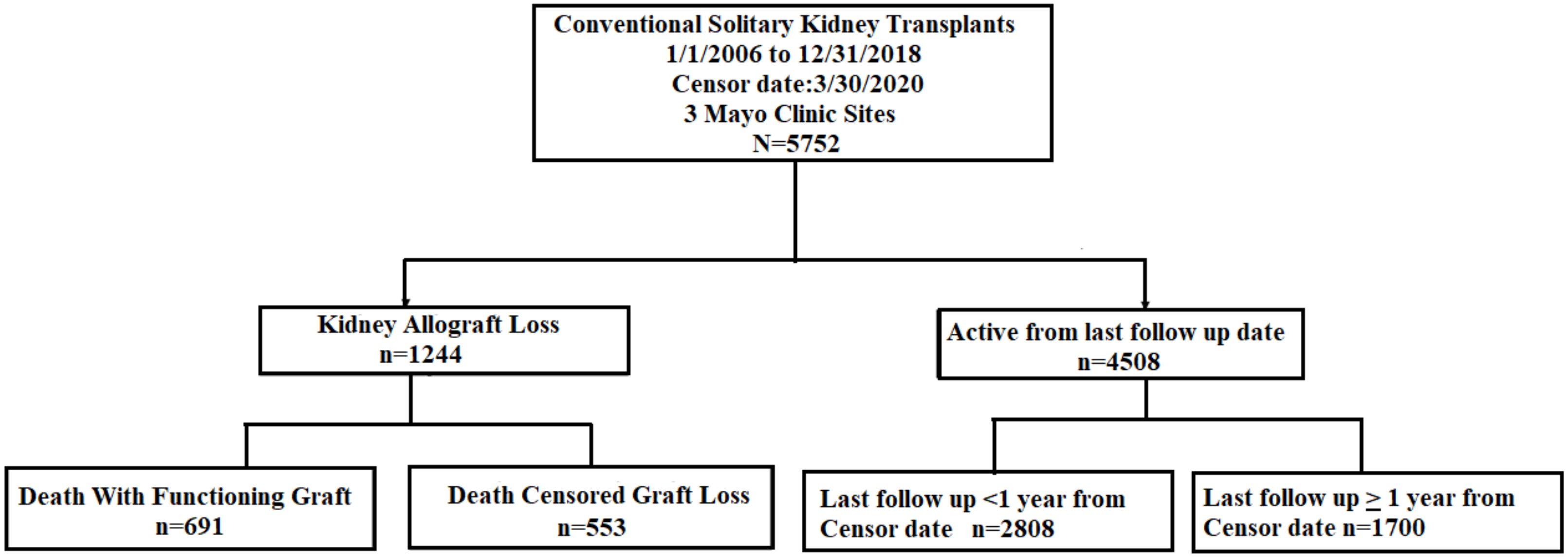

Figure S2. Causes of Graft Loss during time

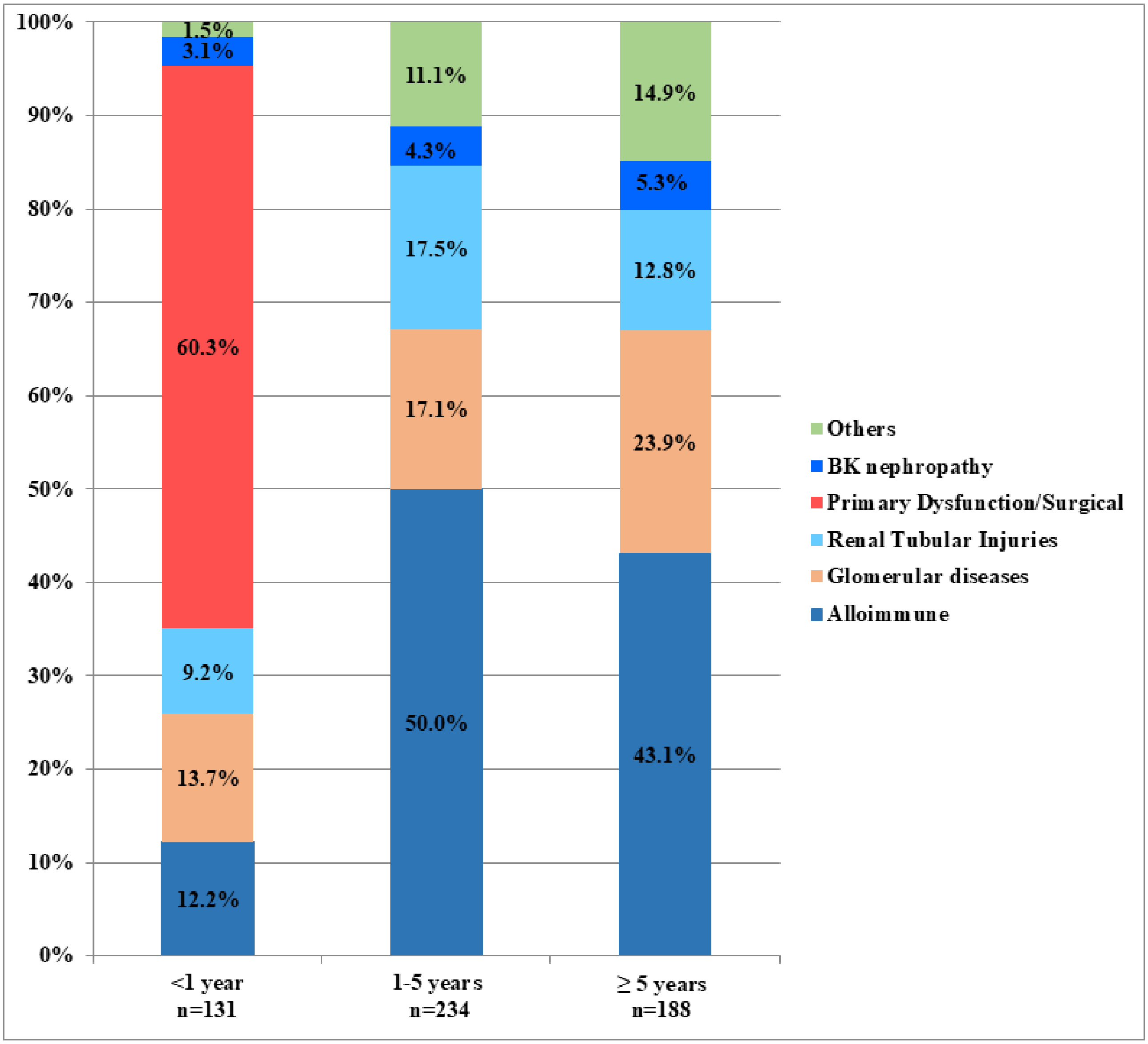

Figure S3. Comparison of Causes of Allograft Failure in Two Eras.

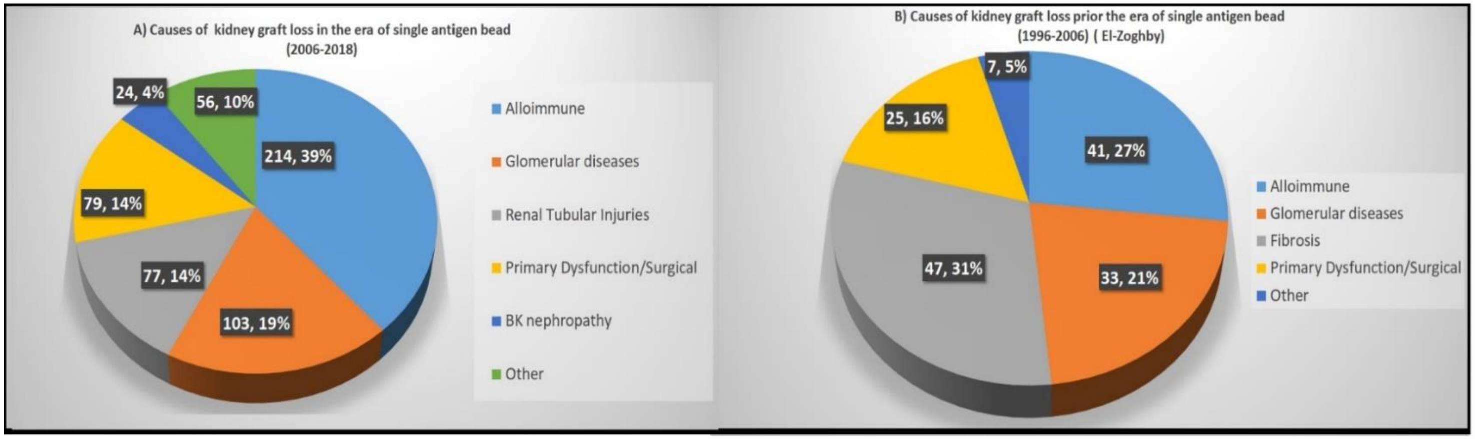

A) Causes of kidney graft loss 2006-2018.
B) Causes of kidney graft loss, single center 1996-2006. (Reference 10)
